# Supplementary material for: Insect hosts are nutritional landscapes navigated by fungal pathogens
Source: Ecology. 2025 Feb 7;106(2):e70015. doi: 10.1002/ecy.70015 (PMC11803695; doi:10.1002/ecy.70015)
Supplement: Supplementary file 1 — Appendix S1: [file ECY-106-e70015-s001.pdf]

Appendix S1

Journal: Ecology

Insect hosts are nutritional landscapes navigated by fungal pathogens

AUTHORS

Henrik H. De Fine Licht, Zsuzsanna Csontos, Piet Jan Domela Nijegaard Nielsen, Enzo Buhl Langkilde, August K. Kjærgård Hansen, Jonathan Z. Shik

Table S1. Isolates of *Metarhizium* used in current study. Isolates in bold were included in the nutritional consumption experiment.

Species	Code	Isolate	Source	Country	Year	Notes
M. anisopliae	**A1**	**ARSEF_549**	**n.a.**	**Brazil**	**1980**	
M. anisopliae	A	ESALQ_1116	*Scarabaeidae*	Brazil	1993	
M. anisopliae	B	ESALQ_1604	n.a.	Brazil	n.a.	Biotech G, Biotech® Controle Biológico (Isolated from commercial isolate)
M. acridum	**B1**	**ARSEF_324**	***Austracris guttulosa***	**Australia**	**1979**	
M. acridum	C	ARSEF_7486	*Ornithacris cavroisi*	Niger	1992	Active ingredient in commercial product *Green Muscle*™
M. acridum	D	ARSEF_3609	*Patanga succincta*	Thailand	1992	
M. robertsii	**E**	**KVL_12-35**	**Soil**	**Denmark**	**2012**	
M. robertsii	F	KVL_13-12	Soil	Denmark	2013	

Table S2. Agar media recipes for 36 fungal diets. All quantities are in grams (g) and total volume of media is 500 mL (Modified from Shik et al. 2016).

P:C	Bacto Peptone	Trypticase Peptone	Bacto Tryptone	Sucrose	Starch	Agar	Vitamin
**Media concentration 4 g/l**							
16:1	0.6676	0.6680	0.6759	0.0539	0.0539	8	0.04
8:1	0.6305	0.6309	0.6383	0.1065	0.1065	8	0.04
5:1	0.5911	0.5915	0.5984	0.1623	0.1623	8	0.04
3:1	0.5320	0.5323	0.5385	0.2460	0.2460	8	0.04
1:1	0.3548	0.3550	0.3590	0.4975	0.4975	8	0.04
1:3	0.1773	0.1775	0.1795	0.7488	0.7488	8	0.04
1:5	0.1182	0.1183	0.1197	0.8325	0.8325	8	0.04
1:8	0.0788	0.0789	0.0798	0.8883	0.8883	8	0.04
1:16	0.0417	0.0418	0.0422	0.9409	0.9409	8	0.04
**Media concentration 8 g/l**							
16:1	1.3352	1.3360	1.3517	0.1079	0.1079	8	0.08
8:1	1.2610	1.2618	1.2766	0.2130	0.2130	8	0.08
5:1	1.1822	1.1829	1.1969	0.3247	0.3247	8	0.08
3:1	1.0640	1.0646	1.0772	0.4922	0.4922	8	0.08
1:1	0.7095	0.7100	0.7180	0.9950	0.9950	8	0.08
1:3	0.3545	0.3550	0.3590	1.4975	1.4975	8	0.08
1:5	0.2364	0.2366	0.2394	1.6649	1.6649	8	0.08
1:8	0.1576	0.1577	0.1596	1.7766	1.7766	8	0.08
1:16	0.0835	0.0835	0.0845	1.8817	1.8817	8	0.08
**Media concentration 20 g/l**							
16:1	3.3381	3.3401	3.3794	0.2697	0.2697	8	0.2
8:1	3.1526	3.1545	3.1916	0.5325	0.5325	8	0.2
5:1	2.9556	2.9573	2.9921	0.8117	0.8117	8	0.2
3:1	2.6600	2.6615	2.6930	1.2305	1.2305	8	0.2
1:1	1.7735	1.7745	1.7955	2.4870	2.4870	8	0.2
1:3	0.8865	0.8870	0.8975	3.7435	3.7435	8	0.2
1:5	0.5911	0.5915	0.5984	4.1623	4.1623	8	0.2
1:8	0.3941	0.3943	0.3990	4.4416	4.4416	8	0.2
1:16	0.2086	0.2088	0.2112	4.7044	4.7044	8	0.2
**Media concentration 50 g/l**							
16:1	8.3452	8.3502	8.4484	0.6742	0.6742	8	0.5
8:1	7.8816	7.8863	7.9790	1.3312	1.3312	8	0.5
5:1	7.3890	7.3934	7.4803	2.0293	2.0293	8	0.5
3:1	6.6500	6.6538	6.7325	3.0763	3.0763	8	0.5
1:1	4.4331	4.4331	4.4881	6.2175	6.2175	8	0.5
1:3	2.2169	2.2181	2.2444	9.3588	9.3588	8	0.5
1:5	1.4778	1.4787	1.4961	10.406	10.406	8	0.5
1:6	0.9852	0.9858	0.9974	11.104	11.104	8	0.5
1:16	0.5216	0.5219	0.5280	11.761	11.761	8	0.5

Table S3. Eight-scale categories of fungus colony colour changes in *Metarhizium* fungi as a measure of conidia formation. Example pictures are shown in Supplementary Figure S3.

Category	Pigmentation
0	Exploratory growth
1	White
2	Light yellow (Beige)
3	Yellow
4	Dark yellow (Orange)
5	Yellow and light green spots
6	Light green
7	Green
8	Dark green (Brownish)

Table S4. Regression analyses supporting heatmaps of mean species cultivar growth area (mm^2), color category (green color representing onset of sporulation), and spore number (log_{10} spore number). Presented are least square regression significances for both linear and quadratic terms for protein (P) and carbohydrate (C) composition in agar plate substrates, as well as the P x C interaction. Significant results for univariate models support the overall interpretation of FNN heatmaps visualizing fungal growth area (mm^2) across the 36 protein and carbohydrate combinations. The across-isolate (species mean) analyses are based on mean-value analysis, averaging across the P:C treatment means for each isolate, with three isolates for *M. anisopliae* ESALQ_1116, ESALQ_1604, ARSEF_549), three isolates for *M. acridum* (ARSEF_7486, ARSEF_3609, ARSEF_324), and two isolates for *M. robertsii* (KVL_12-35, KVL_13-12). The same analyses were also performed at the individual isolate level (Table S5). See Methods, Table S2 and S3, and Figure S3 for more information about how growth area, color category, and spore number were calculated.

**Significance test for univariate models**					
**Model**	**DF model**	**DF error**	**R^2 adjusted**	**F**	***P***
M. anisopliae					
Area	5	30	0.87	47.04	**< 0.0001**
Color category	5	30	0.79	26.69	**< 0.0001**

Spore number	5	30	0.82	32.96	**< 0.0001**
M. acridum					
Area	5	30	0.78	25.23	**< 0.0001**
Color	5	30	0.79	27.79	**< 0.0001**
Spore number	5	30	0.44	6.46	**0.0003**
M. robertsii					
Area	5	30	0.81	31.37	**0.0001**
Color	5	30	0.41	5.93	**0.0006**
Spore	5	30	0.39	5.51	**0.001**

**Univariate tests of Parameter estimates**				
**Isolate**	**Parameter**	**DF**	**F**	**P**
M. anisopliae				
Area	P	1	117.84	**< 0.0001**
	C	1	0.55	0.464
	P^2	1	66.30	**< 0.0001**
	C^2	1	2.58	0.119
	PC	1	7.02	**0.013**
	Error	30		
Color	P	1	33.35	**< 0.0001**
	C	1	15.28	**0.0005**
	P^2	1	9.48	**0.004**
	C^2	1	7.04	**0.013**
	PC	1	0.05	0.821
	Error	30		
Spore number	P	1	10.72	**0.001**
	C	1	23.95	**< 0.0001**
	P^2	1	0.44	0.741
	C^2	1	0.07	0.603
	PC	1	20.71	**< 0.0001**
	Error	30		

M. acridum				
Area	P	1	85.16	**< 0.0001**
	C	1	0.78	0.384
	P^2	1	61.53	**< 0.0001**
	C^2	1	2.84	0.102
	PC	1	6.57	**0.016**
	Error	30		
Color	P	1	23.06	**< 0.0001**
	C	1	5.78	**0.023**
	P^2	1	14.89	**0.0006**
	C^2	1	6.80	**0.014**
	PC	1	17.74	**0.0002**
	Error	30		
Spore number	P	1	0.98	0.329
	C	1	14.43	**0.0007**
	P^2	1	0.09	0.769
	C^2	1	4.00	0.055
	PC	1	1.63	0.212
	Error	30		
M. robertsii				
Area	P	1	124.21	**< 0.0001**
	C	1	3.87	0.058
	P^2	1	77.87	**< 0.0001**
	C^2	1	0.43	0.516
	PC	1	0.07	0.792
	Error	30		
Color	P	1	4.30	**0.047**
	C	1	3.37	0.076
	P^2	1	2.23	0.145
	C^2	1	2.31	0.139
	PC	1	1.78	0.192

	Error	30		
Spore number	P	1	7.38	**0.011**
	C	1	13.16	**0.001**
	P^2	1	3.39	0.076
	C^2	1	5.74	**0.023**
	PC	1	1.37	0.251
	Error	30		
Parameter estimates				
Isolate	Parameter		Estimate	**P**
M. anisopliae				
Area	P		0.40	**< 0.0001**
	C		0.08	**0.038**
	P^2		-0.01	**< 0.0001**
	C^2		-0.00	0.119
	PC		-0.00	**0.013**
Color	P		-0.33	**< 0.0001**
	C		-0.22	**0.005**
	P^2		0.00	**0.004**
	C^2		0.00	**0.013**
	PC		-0.00	0.820
Spore number	P		0.08	0.100
	C		0.02	0.697
	P^2		-0.00	0.513
	C^2		-0.00	0.787
	PC		-0.01	**< 0.0001**
M. acridum				
Area	P		0.16	**< 0.0001**
	C		0.04	**0.033**
	P^2		-0.00	**< 0.0001**
	C^2		-0.00	0.102
	PC		-0.00	**0.016**

Color	P	-0.46	**< 0.0001**
	C	-0.32	**0.0001**
	P^2	0.01	**0.0006**
	C^2	0.00	**0.014**
	PC	0.01	**0.0002**
Spore number	P	-0.00	0.967
	C	-0.18	**0.028**
	P^2	0.00	0.770
	C^2	0.00	0.055
	PC	-0.00	0.212
M. robertsii			
Area	P	0.49	**< 0.0001**
	C	-0.08	0.164
	P^2	-0.01	**< 0.0001**
	C^2	0.00	0.516
	PC	-0.00	0.792
Color	P	-0.24	**0.020**
	C	-0.22	**0.031**
	P^2	0.00	0.146
	C^2	0.00	0.139
	PC	0.01	0.192
Spore number	P	-0.18	0.146
	C	-0.27	**0.034**
	P^2	0.00	0.076
	C^2	0.01	**0.023**
	PC	-0.01	0.251

Table S5. Regression analyses supporting heatmaps of individual isolate cultivar growth area (mm^2), color category, and spore number (log_{10} spore number). Presented are least square regression significances for both linear and quadratic terms for protein (P) and carbohydrate (C) composition in agar plate substrates, as well as the P x C interaction. Significant results for univariate models support the overall interpretation of FNN heatmaps visualizing fungal growth area (mm^2) across the 36 protein and carbohydrate combinations. We provide separate analyses of each of the 8 isolates (*M. anisopliae* (ESALQ_1116, ESALQ_1604, ARSEF_549), *M. acridum* (ARSEF_7486, ARSEF_3609, ARSEF_324), *M. robertsii* (KVL_12-35, KVL_13-12). See Methods, Table S2 and S3, and Figure S3 for more information about how growth area, color, and spore number were calculated.

**Significance test for univariate models**					
**Model**	**DF model**	**DF error**	**R^2 adjusted**	**F**	***P***
M. anisopliae [ESALQ_1116]					
Area	5	30	0.84	37.81	**< 0.0001**
Color	5	30	0.79	27.45	**< 0.0001**
Spore number	5	30	0.76	22.58	**< 0.0001**
M. anisopliae [ESALQ_1604]					
Area	5	30	0.59	10.98	**< 0.0001**
Color	5	30	0.59	10.87	**< 0.0001**
Spore number	5	30	0.07	1.56	0.201
M. anisopliae [ARSEF_549]					
Area	5	30	0.79	27.74	**< 0.0001**
Color	5	30	0.91	76.19	**< 0.0001**
Spore number	5	30	0.81	30.29	**< 0.0001**
M. acridum [ARSEF_7486]					
Area	5	30	0.75	21.61	**< 0.0001**
Color	5	30	0.74	20.92	**< 0.0001**
Spore number	5	30	0.26	3.42	**< 0.0001**
M. acridum [ARSEF_3609]					
Area	5	30	0.88	54.34	**< 0.0001**
Color	5	30	0.53	8.90	**< 0.0001**

Spore number	5	30	0.55	9.61	**< 0.0001**
M. acridum [ARSEF_324]					
Area	5	30	0.38	5.23	**0.001**
Color	5	30	0.84	37.55	**< 0.0001**
Spore number	5	30	0.11	1.85	0.134
M. robertsii [KVL_12-35]					
Area	5	30	0.47	7.24	**0.0001**
Color	5	30	0.25	3.34	**0.016**
Spore	5	30	0.56	9.87	**< 0.0001**
M. robertsii [KVL_13-12]					
Area	5	30	0.88	51.96	**< 0.0001**
Color	5	30	0.47	7.18	**0.0002**
Spore number	5	30	0.21	2.81	**0.033**

**Univariate tests of Parameter estimates**				
**Isolate**	**Parameter**	**DF**	**F**	**P**
M. anisopliae [ESALQ_1116]				
Area	P	1	83.34	**< 0.0001**
	C	1	0.28	0.599
	P^2	1	45.99	**< 0.0001**
	C^2	1	1.08	0.308
	PC	1	7.95	**0.008**
	Error	30		
Color	P	1	64.95	**< 0.0001**
	C	1	4.62	**0.040**
	P^2	1	31.26	**< 0.0001**
	C^2	1	3.91	0.057
	PC	1	0.61	0.439
	Error	30		
Spore number	P	1	12.67	**0.001**
	C	1	21.25	**< 0.0001**

	P^2	1	0.11	0.741
	C^2	1	0.28	0.603
	PC	1	59.20	**< 0.0001**
	Error	30		
M. anisopliae [ESALQ_1604]				
Area	P	1	29.29	**< 0.0001**
	C	1	3.69	0.064
	P^2	1	14.01	**0.0008**
	C^2	1	1.41	0.245
	PC	1	0.08	0.786
	Error	30		
Color	P	1	7.20	**0.012**
	C	1	13.16	**0.001**
	P^2	1	1.63	0.212
	C^2	1	7.046	**0.013**
	PC	1	0.015	0.903
	Error	30		
Spore number	P	1	0.01	0.916
	C	1	0.63	0.433
	P^2	1	0.07	0.79
	C^2	1	0.12	0.732
	PC	1	0.490	0.489
	Error	30		
M. anisopliae [ARSEF_549]				
Area	P	1	57.79	**< 0.0001**
	C	1	10.63	**< 0.0001**
	P^2	1	36.17	**0.003**
	C^2	1	9.03	**0.005**
	PC	1	5.30	**0.028**
	Error	30		
Color	P	1	43.41	**< 0.0001**

	C	1	25.29	**< 0.0001**
	P^2	1	3.07	0.090
	C^2	1	2.10	0.157
	PC	1	2.65	0.114
	Error	30		
Spore number	P	1	8.03	**0.008**
	C	1	12.95	**0.001**
	P^2	1	1.46	0.237
	C^2	1	1.84	**0.186**
	PC	1	50.86	**< 0.0001**
	Error	30		
M. acridum [ARSEF_7486]				
Area	P	1	88.40	**< 0.0001**
	C	1	2.63	0.116
	P^2	1	55.63	**< 0.0001**
	C^2	1	0.54	0.466
	PC	1	0.08	0.778
	Error	30		
Color	P	1	29.05	**< 0.0001**
	C	1	4.55	**0.041**
	P^2	1	19.10	**0.0001**
	C^2	1	6.69	**0.015**
	PC	1	11.44	**0.002**
	Error	30		
Spore number	P	1	0.060	0.809
	C	1	7.68	**0.009**
	P^2	1	0.85	0.365
	C^2	1	1.24	0.275
	PC	1	4.98	**0.033**
	Error	30		

M. acridum [ARSEF_3609]				
Area	P	1	97.99	**< 0.0001**
	C	1	15.19	**0.0005**
	P^2	1	44.61	**< 0.0001**
	C^2	1	14.10	**0.0007**
	PC	1	3.54	**0.070**
	Error	30		
Color	P	1	19.90	**0.018**
	C	1	0.090	0.868
	P^2	1	8.890	0.106
	C^2	1	0.153	0.828
	PC	1	15.26	**0.037**
	Error	30		
Spore number	P	1	5.10	**0.031**
	C	1	10.93	**0.002**
	P^2	1	1.76	0.195
	C^2	1	0.90	0.349
	PC	1	4.64	**0.039**
	Error	30		
M. acridum [ARSEF_324]				
Area	P	1	16.14	**0.0004**
	C	1	0.24	0.626
	P^2	1	18.76	**0.0002**
	C^2	1	1.42	0.243
	PC	1	6.63	**0.015**
	Error	30		
Color	P	1	17.01	**0.0003**
	C	1	21.71	**< 0.0001**
	P^2	1	14.05	**0.0008**
	C^2	1	17.63	**0.0002**
	PC	1	25.92	**< 0.0001**

	Error	30		
Spore number	P	1	0.09	0.765
	C	1	6.07	**0.020**
	P^2	1	0.63	0.630
	C^2	1	0.06	0.062
	PC	1	0.91	0.912
	Error	30		
M. robertsii [KVL_12-35]				
Area	P	1	28.68	**< 0.0001**
	C	1	0.01	0.927
	P^2	1	16.96	**0.0003**
	C^2	1	0.05	0.824
	PC	1	1.35	0.254
	Error	30		
Color	P	1	1.36	0.253
	C	1	1.48	0.233
	P^2	1	0.05	0.833
	C^2	1	0.25	0.620
	PC	1	0.05	0.832
	Error	30		
Spore number	P	1	6.06	**0.020**
	C	1	18.70	**0.0002**
	P^2	1	1.08	0.304
	C^2	1	3.72	0.063
	PC	1	7.61	**0.010**
	Error	30		
M. robertsii [KVL_13-12]				
Area	P	1	183.67	**< 0.0001**
	C	1	12.87	**0.001**
	P^2	1	118.86	**< 0.0001**

	C^2	1	2.42	0.130
	PC	1	4.55	**0.041**
	Error	30		
Color	P	1	6.50	**0.016**
	C	1	4.21	**0.049**
	P^2	1	6.40	**0.017**
	C^2	1	5.10	**0.031**
	PC	1	7.31	**0.011**
	Error	30		
Spore number	P	1	6.41	**0.017**
	C	1	6.07	**0.020**
	P^2	1	5.15	**0.031**
	C^2	1	6.00	**0.020**
	PC	1	0.l7	0.682
	Error	30		
**Parameter estimates**				
**Isolate**	**Parameter**		**Estimate**	**P**
M. anisopliae [ESALQ_1116]				
Area	P		0.44	**< 0.0001**
	C		-0.01	**< 0.0001**
	P^2		0.06	0.223
	C^2		-0.00	0.308
	PC		-0.01	**0.008**
Color	P		-0.55	**< 0.0001**
	C		-0.17	**0.036**
	P^2		0.01	**< 0.0001**
	C^2		0.00	0.057
	PC		0.00	0.439
Spore number	P		0.12	0.100
	C		0.06	0.402
	P^2		-0.00	0.741

	C^2	0.00	0.603
	PC	-0.02	**< 0.0001**
M. anisopliae [ESALQ_1604]			
Area	P	0.25	**< 0.0001**
	C	-0.08	0.177
	P^2	-0.00	0.001
	C^2	0.00	0.245
	PC	-0.00	0.786
Color	P	-0.25	**0.033**
	C	-0.33	**0.005**
	P^2	0.00	0.211
	C^2	0.01	0.013
	PC	0.00	0.903
Spore number	P	0.12	0.100
		0.06	0.402
	P^2	-0.00	0.741
	C^2	0.00	0.603
	PC	-0.02	**< 0.0001**
M. anisopliae [ARSEF_549]			
Area	P	0.50	**< 0.0001**
	C	0.27	**0.0004**
	P^2	-0.01	**< 0.0001**
	C^2	0.00	**0.005**
	PC	-0.01	**0.028**
Color	P	0.05	**0.0001**
	C	0.05	**0.004**
	P^2	0.00	0.090
	C^2	0.00	0.157
	PC	0.00	0.114
Spore number	P	0.17	0.061
	C	0.12	0.193

	P^2	-0.00	0.237
	C^2	-0.00	0.186
	PC	-0.02	**< 0.0001**
M. acridum [ARSEF_7486]			
Area	P	0.15	**< 0.0001**
	C	-0.02	0.265
	P^2	-0.00	**< 0.0001**
	C^2	0.00	0.466
	PC	-0.00	0.778
Color	P	-0.53	**0.0001**
	C	-0.31	**0.0008**
	P^2	0.01	**0.0001**
	C^2	0.00	**0.015**
	PC	0.01	**0.002**
Spore number	P	0.03	0.139
	C	-0.02	0.371
	P^2	-0.00	0.365
	C^2	0.00	0.275
	PC	-0.00	**0.033**
M. acridum [ARSEF_3609]			
Area	P	0.14	**< 0.0001**
	C	0.07	**0.0002**
	P^2	-0.00	**< 0.0001**
	C^2	-0.00	**0.0007**
	PC	-0.00	0.070
Color	P	-0.36	**0.002**
	C	-0.12	0.256
	P^2	0.00	0.106
	C^2	0.00	0.828
	PC	0.01	**0.037**
Spore number	P	0.03	0.139

	C	-0.02	0.371
		-0.00	0.365
	C^2	0.00	0.275
	PC	-0.00	**0.033**
M. acridum [ARSEF_324]			
Area	P	0.19	**< 0.0001**
	C	0.08	**< 0.0001**
	P^2	-0.00	0.064
	C^2	-0.00	**0.0002**
	PC	-0.00	**0.015**
Color	P	-0.36	**0.002**
	C	-0.12	0.256
	P^2	0.00	0.106
	C^2	0.00	0.828
	PC	0.01	**0.037**
Spore number	P	0.03	0.861
	C	-0.34	**0.049**
	P^2	-0.00	0.630
	C^2	0.01	0.062
	PC	0.00	0.912
M. robertsii [KVL_12-35]			
Area	P	0.29	**0.001**
	C	-0.06	0.451
	P^2	-0.01	**0.0003**
	C^2	-0.00	0.824
	PC	0.00	0.254
Color	P	-0.10	0.424
	C	-0.10	0.400
	P^2	0.00	0.833
	C^2	0.00	0.620
	PC	-0.00	0.832

Spore number	P	-0.04	0.731
	C	-0.23	0.074
	P^2	0.00	0.304
	C^2	0.00	0.063
	PC	-0.01	**0.010**
M. robertsii [KVL_13-12]			
Area	P	0.69	**< 0.0001**
	C	-0.09	0.4115
	P^2	-0.01	**< 0.0001**
	C^2	0.00	0.130
	PC	-0.00	**0.041**
Color	P	-0.39	**0.001**
	C	-0.35	**0.003**
	P^2	0.01	**0.017**
	C^2	0.00	**0.031**
	PC	0.01	**0.011**
Spore number	P	-0.04	0.731
	C	-0.23	0.074
	P^2	0.00	0.304
	C^2	0.00	0.063
	PC	-0.01	**0.010**

Table S6. Linear regression analysis comparing fungal nutritional consumption in relation to the ratio of protein and carbohydrate in the media. The results of comparing the difference between coefficients of the linear model of *lm*(carbohydrates~protein) with the H_0 of a linear consumption of media ratios (either 3.00 or 0.33). Tests were conducted using the function *LinearHypothesis* from the *car* package (Fox & Weisberg, 2019) in R version 4.3.1.

Media composition	Slope estimate	H_0	F	*p*
M. anisopliae				
15 g/L, P:C = 1:3	2.15	3.00	2.3940	0.1528
15 g/L, P:C = 3:1	0.11	0.33	**6.3541**	**0.0304**
50 g/L, P:C = 1:3	1.54	3.00	2.9302	0.1177
50 g/L, P:C = 3:1	0.37	0.33	0.2295	0.6433
M. robertsii				
15 g/L, P:C = 1:3	0.30	3.00	**29.982**	**0.0003**
15 g/L, P:C = 3:1	0.22	0.33	3.3850	0.0956
50 g/L, P:C = 1:3	0.84	3.00	**19.646**	**0.0013**
50 g/L, P:C = 3:1	0.40	0.33	0.3192	0.5846
M. acridum				
15 g/L, P:C = 1:3	2.02	3.00	3.4500	0.0929
15 g/L, P:C = 3:1	0.20	0.33	1.1641	0.3060
50 g/L, P:C = 1:3	1.72	3.00	3.5239	0.0899
50 g/L, P:C = 3:1	0.36	0.33	0.0282	0.8700

Fox, J. and Weisberg, S. (2019) An R Companion to Applied Regression, Third Edition, Sage.

Supplementary figures

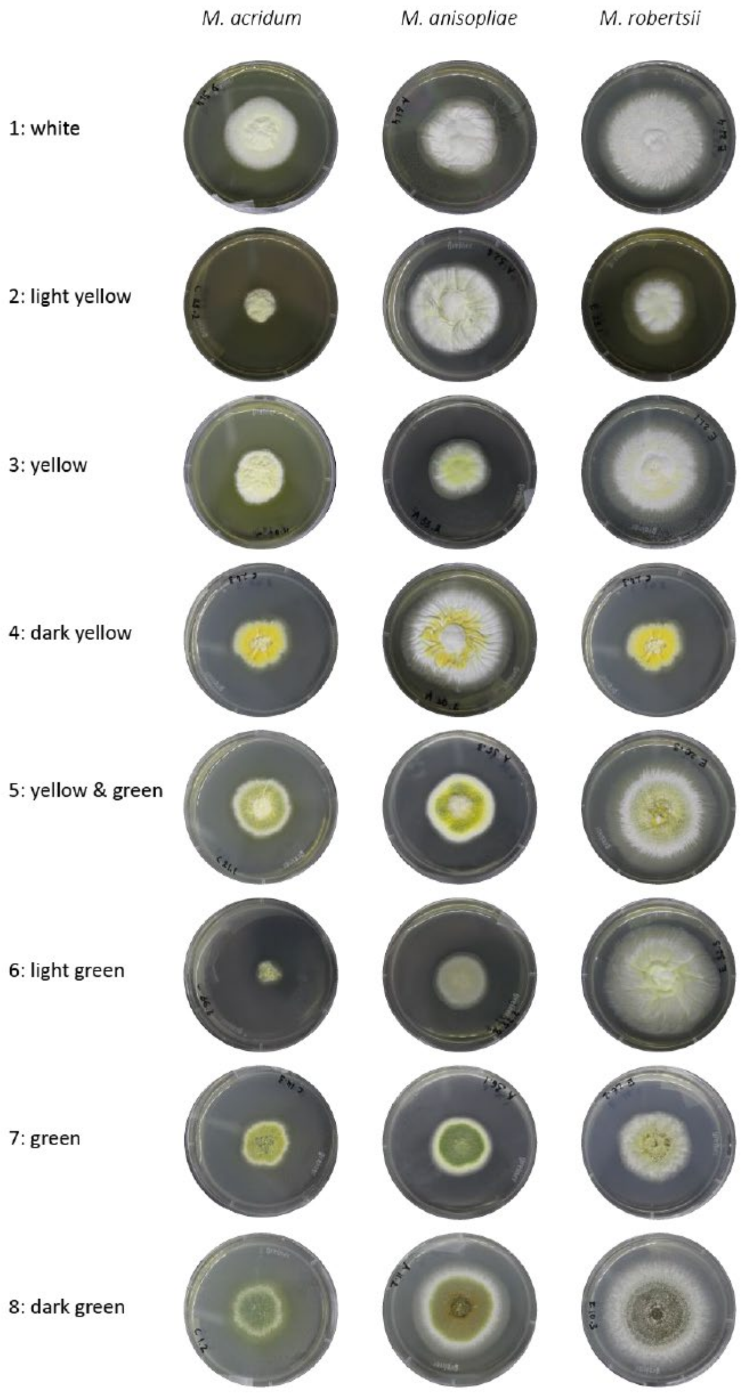

Figure S1. **Colony pigmentation scoring categories of *Metarhizium* fungi.** All Photos: Zsuzsanna Csontos.

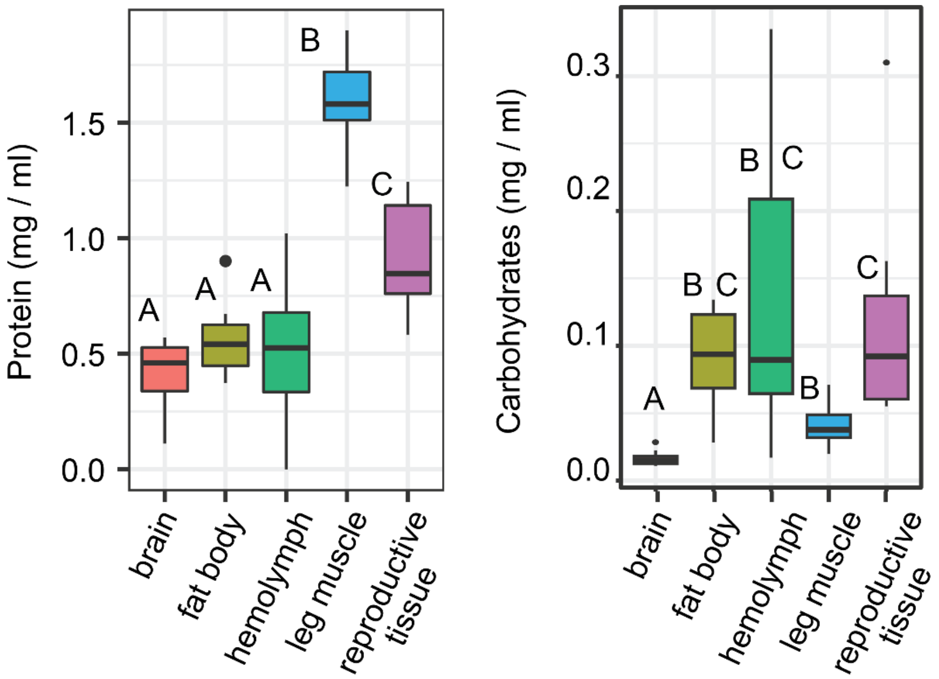

Figure S2. Total protein and carbohydrate measurements of specific insect tissues. Letters above each boxplot represents significant group differences following GLM analysis. Solid line inside boxplots represent the median, the box the interquartile 50% of the data, and whiskers represent the distance between maximum and minimum values to the interquartile range, and separate dots are outliers.
